# Endoscopic repair of a bronchoesophageal fistula: a dual approach with argon plasma coagulation and clips with anchoring prongs

**DOI:** 10.1055/a-2760-8933

**Published:** 2025-12-17

**Authors:** Xenofon Tsamakidis, Alexandros Ioannou, Athanasios Kontos, Dionysia Mandilara, Ioannis Tziortziotis, Dimitris Dimitroulopoulos, Dimitrios Kypraios

**Affiliations:** 1 69068 Department of Gastroenterology, Agios Savvas General Cancer and Oncology Hospital of Athens, Athens, Greece; 2 37781 Department of Gastroenterology, General Hospital of Athens Alexandra, Athens, Greece

Management of tracheoesophageal and bronchoesophageal fistulas remains highly challenging, as surgical repair is associated with significant morbidity 1 . Endoscopic alternatives such as stenting 1 2 , over-the-scope clips 1 3 and glue injection 4 have been employed, though with limited long-term success.

We present the case of a 44-year-old man with mild dysphagia and postprandial coughing, reporting recurrent lower respiratory tract infections over recent months. His medical history included the surgical repair of esophageal atresia during the neonatal period and endoscopic bougie dilations at age 17 years due to a benign stricture at the anastomotic site.

A water-soluble contrast esophagogram revealed leakage into a left bronchus, consistent with a bronchoesophageal fistula ( Fig. 1 ). Gastroscopy identified four fistula orifices at the mid-esophageal anastomosis ( Fig. 2 ). Initial closure using conventional through-the-scope clips was unsuccessful due to slippage over the fibrotic tissue.

**Fig. 1:**
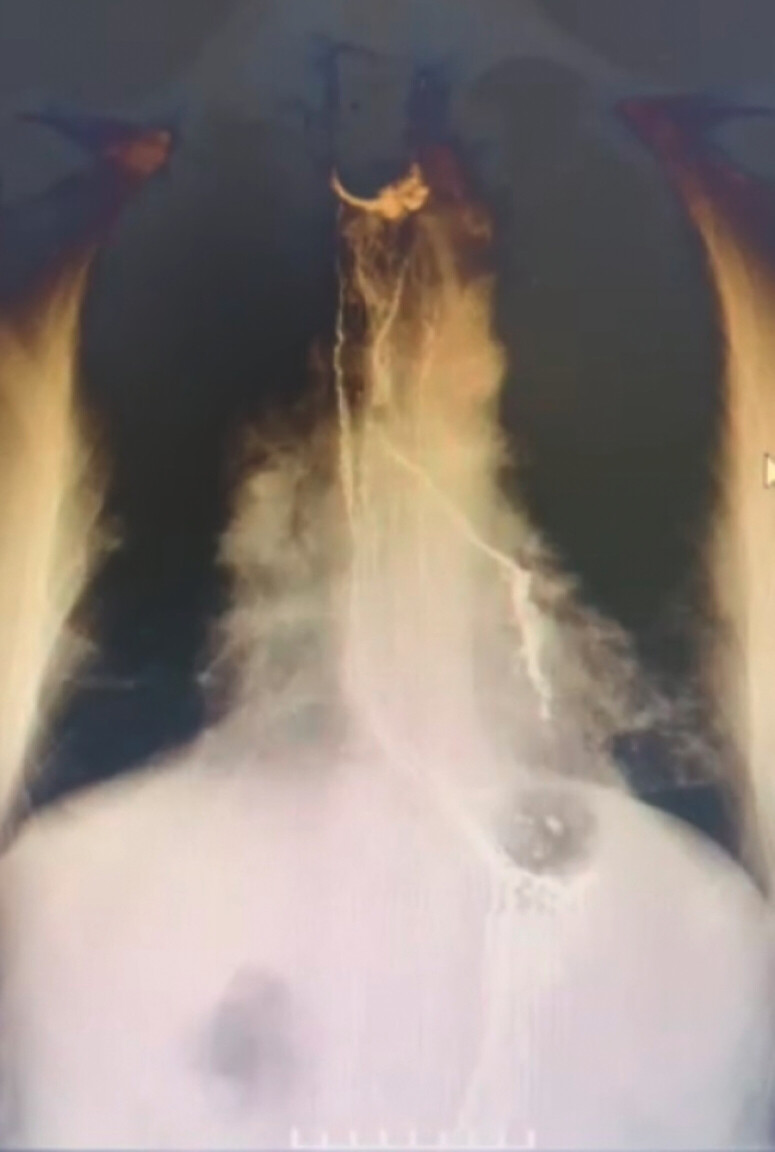
Water-soluble contrast esophagogram demonstrating a bronchoesophageal fistula.

**Fig. 2:**
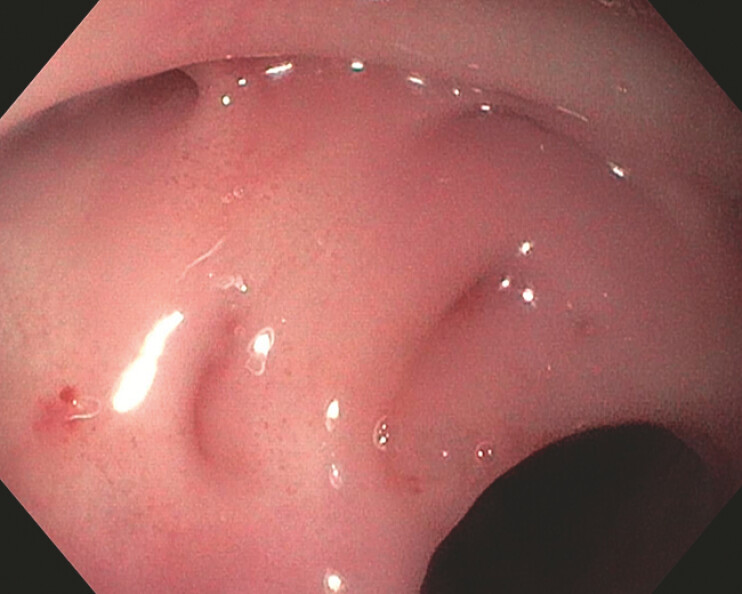
Endoscopic view showing four fistula orifices at the site of the esophageal anastomosis.

Argon plasma coagulation (APC) was applied to the tissue surrounding the orifices to enhance tissue preparation ( Fig. 3 ), followed by the placement of three clips with anchoring prongs (MANTIS; Fig. 4 ), resulting in successful fistula closure ( Video 1 ). A follow-up esophagogram after 1 month confirmed the absence of leakage ( Fig. 5 ), while at 4 months the patient reported complete resolution of coughing and respiratory infections.

**Fig. 3:**
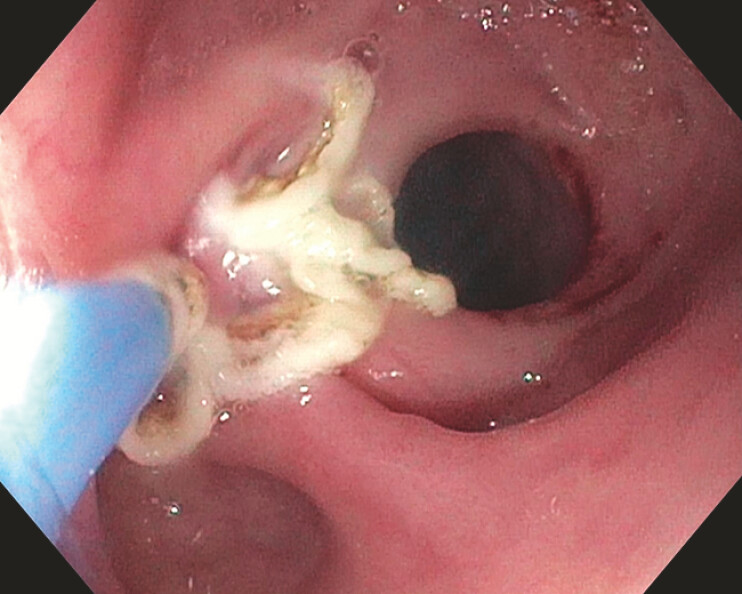
APC applied to the mucosa surrounding the fistula orifices. APC, argon plasma coagulation.

**Fig. 4:**
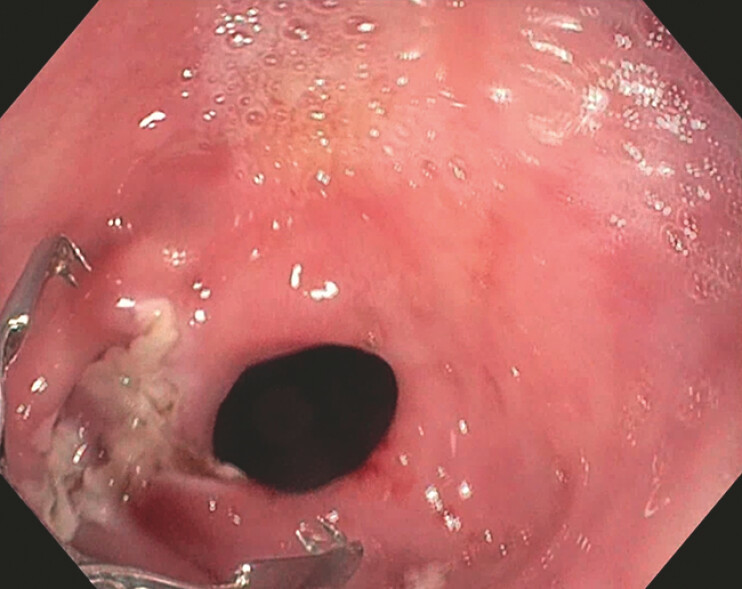
Closure of the fistula orifices using MANTIS clips.

Combined application of argon plasma coagulation and MANTIS clips for endoscopic closure of a benign bronchoesophageal fistula.Video 1

**Fig. 5:**
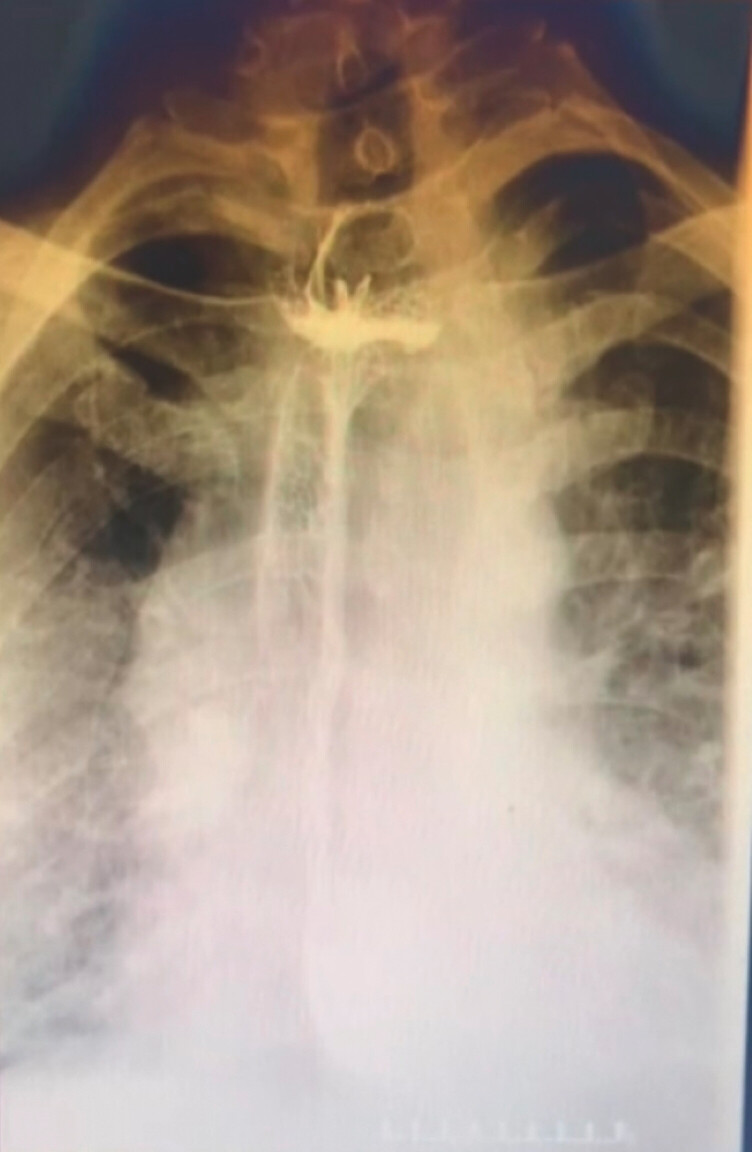
A post-procedural water-soluble contrast esophagogram confirming successful fistula closure without leakage.

MANTIS clips offer improved grip and deeper anchoring in fibrotic or scarred tissues due to their serrated jaws, reducing the risk of slippage and enabling more secure closure. APC cauterization is believed to enhance clip adherence and facilitate fistula healing by promoting tissue de-epithelialization 5 .

This case demonstrates a minimally invasive, effective approach for complex benign bronchoesophageal fistulas. To our knowledge, this is the first reported case utilizing the combined application of APC and MANTIS clips for this indication, supporting its potential as a safe and promising alternative to surgery in selected patients.

Endoscopy_UCTN_Code_TTT_1AO_2AI

## Notes

Endoscopy E-Videos https://eref.thieme.de/e-videos
*E-Videos* is an open access online section of the journal *Endoscopy* , reporting on interesting cases and new techniques in gastroenterological endoscopy. All papers include a high-quality video and are published with a Creative Commons CC-BY license. Endoscopy E-Videos qualify for HINARI discounts and waivers and eligibility is automatically checked during the submission process. We grant 100% waivers to articles whose corresponding authors are based in Group A countries and 50% waivers to those who are based in Group B countries as classified by Research4Life (see: https://www.research4life.org/access/eligibility/ ). This section has its own submission website at https://mc.manuscriptcentral.com/e-videos .
